# Efficient Regulation of Polysulfides by Anatase/Bronze TiO_2_ Heterostructure/Polypyrrole Composites for High-Performance Lithium-Sulfur Batteries

**DOI:** 10.3390/molecules28114286

**Published:** 2023-05-23

**Authors:** Jing Liu, Yong Liu, Tengfei Li, Longlong Liang, Sifan Wen, Yue Zhang, Guilong Liu, Fengzhang Ren, Guangxin Wang

**Affiliations:** 1 School of Materials Science and Engineering, Provincial and Ministerial Co-Construction of Collaborative Innovation Center for Non-Ferrous Metal New Materials and Advanced Processing Technology, Henan University of Science and Technology, Luoyang 471023, China; liuliu158600@163.com (J.L.); fei1281156656@163.com (T.L.);; 2 Key Laboratory of Function-Oriented Porous Materials of Henan Province, College of Chemistry and Chemical Engineering, Luoyang Normal University, Luoyang 471934, China

**Keywords:** anatase/bronze TiO_2_ heterostructure, polypyrrole, Li-S batteries, lithium polysulfides immobilization, electrochemical performance

## Abstract

Despite having ultra-high theoretical specific capacity and theoretical energy density, lithium-sulfur (Li-S) batteries suffer from their low Coulombic efficiency and poor lifespan, and the commercial application of Li-S batteries is seriously hampered by the severe “shuttle effect” of lithium polysulfides (LiPSs) and the large volume expansion ratio of the sulfur electrode during cycling. Designing functional hosts for sulfur cathodes is one of the most effective ways to immobilize the LiPSs and improve the electrochemical performance of a Li-S battery. In this work, a polypyrrole (PPy)-coated anatase/bronze TiO_2_ (TAB) heterostructure was successfully prepared and used as a sulfur host. Results showed that the porous TAB could physically adsorb and chemically interact with LiPSs during charging and discharging processes, inhibiting the LiPSs’ shuttle effect, and the TAB’s heterostructure and PPy conductive layer are conducive to the rapid transport of Li^+^ and improve the conductivity of the electrode. By benefitting from these merits, Li-S batteries with TAB@S/PPy electrodes could deliver a high initial capacity of 1250.4 mAh g^−1^ at 0.1 C and show an excellent cycling stability (the average capacity decay rate was 0.042% per cycle after 1000 cycles at 1 C). This work brings a new idea for the design of functional sulfur cathodes for high-performance Li-S battery.

## 1. Introduction

With the high-speed development of electric vehicles and portable electronics, traditional Li-ion batteries have been incapable of meeting people’s growing demands for high-energy-density energy storage systems [1,2]. In this context, the lithium-sulfur (Li-S) battery is deemed to be one of the most promising candidates for next-generation energy storage systems because of their high theoretical energy density (2700 wh kg^−1^) [3], low cost and environmental benignity [4]. However, there are still a number of problems which are severely hindering the commercial application of Li-S batteries, which include the sulfur cathode’s large volume expansion during charge and discharge processes and the severe “shuttle effect” of lithium polysulfides (LiPSs), leading to low Coulombic efficiencies and limited cycling stability [5,6]. To solve the aforementioned problems, various strategies have been explored, mainly including the design of sulfur cathode hosts [7,8], electrolyte optimization [9], separator modification [10,11,12,13] and Li metal anode protection [14,15,16]. Until now, a variety of carbon-based sulfur host materials have been investigated for sulfur hosts, such as graphene [17,18,19], carbon nanotubes [20,21,22], carbon nanofibers [23,24], carbon nanospheres [25,26], porous carbon [27,28,29] and polymer [30], due to their excellent electrical conductivity. However, these pristine carbon materials do not have polarity, they could not chemically absorb LiPSs and it is hard to effectively regulate the “shuttle effect” of LiPSs, resulting in the unsatisfactory electrochemical performance of Li-S batteries [31,32]. In this regard, polar materials have been widely investigated as sulfur hosts in recent years, such as metal oxides [33,34,35], metal sulfides [36], metal nitrides [37], metal carbide [38] and metal hydroxides [39], and these could effectively absorb and immobilize polar polysulfides via chemical interaction [40].

Recently, as one of the most promising metal oxide sulfur host materials, titanium dioxide (TiO_2_) has been extensively investigated as a sulfur host because of the advantages of non-toxicity, adjustable morphology and good polysulfides immobilization ability [41,42]. Among the different allotropes of TiO_2_, anatase TiO_2_ is one of the most commonly used types for the hosts of sulfur cathode, but its electrical conductivity is limited, making it unable to efficiently transport Li^+^ [43]. Furthermore, bronze TiO_2_ is a kind of metastable monoclinic TiO_2_ with an open channel structure, which exhibits special pseudo-capacitive behavior and can accommodate more Li^+^ than anatase TiO_2_ [44]. Moreover, nanocrystals with different bandgaps form heterogeneous structures through coupling, which can not only improve the efficiency of charge transport in the crystal, but also improve the surface reaction kinetics [45]. For instance, TiO_2_-VOx [46], TiN-TiO_2_ [47] and Ti_3_C_2_-TiO_2_ [48] heterostructure sulfur hosts show significant improvement in conductivity and dynamical acceleration on polysulfides conversion. The anatase TiO_2_ and bronze TiO_2_ were reported to be different in band gap [49]. In addition, our previous work reported that charge separation at the anatase and bronze TiO_2_ hetero-structural boundary could facilitate the generation of more lithium storage sites, and the reaction kinetics of interfacial storage processes are faster than in bulk [44,50]. Furthermore, previous works have revealed that a conductive polymer coated with sulfur can physically and chemically adsorb LiPSs [51,52].

Based on the above research, we successfully fabricated a new PPy-coated anatase/bronze TiO_2_ (TAB) heterostructure and investigated it as a sulfur host for Li-S batteries. Various characterizations and tests illustrated that the TAB/PPy composite material could not only provide a sufficient space to accommodate the sulfur’s volume expansion during cycling, but could also physically adsorb and chemically interact with LiPSs, inhibiting the “shuttle effect”. The PPy coated TAB heterojunction structure could improve the electron conductivity and Li ion transport efficiency, and the Li-S battery with the TAB@S/PPy electrode exhibited an excellent rate capability and cycling stability.

## 2. Results and Discussion

**Figure 1:**
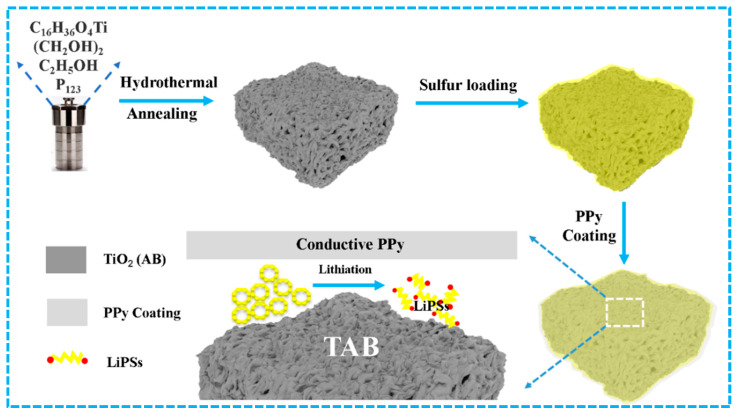
Schematic illustration for the synthesis process of TAB@S/PPy, PPy–polypyrrole, TAB–anatase/bronze TiO_2_, LiPSs–lithium polysulfides.

**Figure 2:**
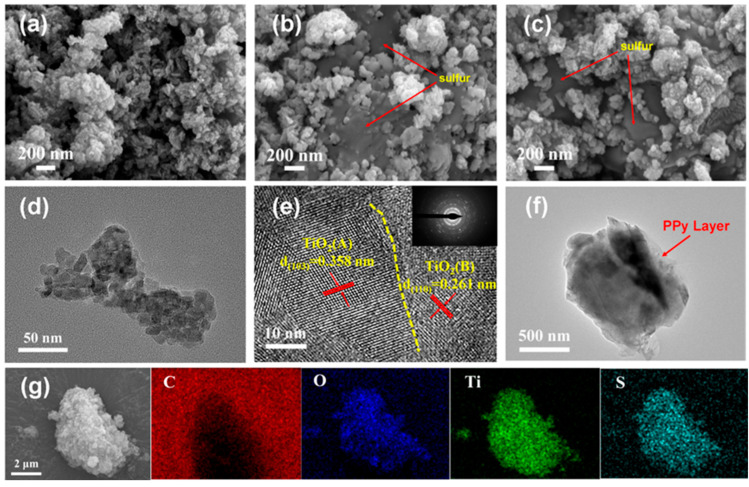
(**a**,**b**) and (**c**) are the SEM images of TAB, TAB@S and TAB@S/PPy, respectively; (**d**,**e**) and (**f**) are the TEM and HRTEM images of TAB, as well as the TEM images of TAB@S/PPy, respectively. The insert image in is the selected area electron diffraction image of TAB; (**g**) the EDS element mapping images of TAB@S/PPy. The color red, blue, green and cyan represent the distribution of C, O, Ti and S elements in TAB@S/PPy, respectively.

The synthetic process of the TAB@S/PPy compound is shown in Figure 1. The TAB material was firstly prepared using the hydrothermal method, followed by an annealing process. The TiO_2_ precursor was obtained after hydrothermal treatment (Figure S1). In the subsequent annealing process, P123 in the sample was gradually evaporated with the increase in temperature, thus forming many pores. As depicted in Figure 2a,d, the scanning electron microscopy (SEM) and transmission electron microscopy (TEM) images showed that the morphology of TAB nanoparticles depicted a sponge structure, and the pores could provide abundant space to accommodate sulfur and relieve the volume expansion of the sulfur cathode during cycling. In Figure 2e, the high-resolution TEM (HRTEM) image of TAB demonstrated the interlayer spacings of 0.3580 nm and 0.261 nm, corresponding to the (103) lattice plane of TiO_2_ (A) and the (110) lattice plane of TiO_2_ (B), respectively, indicating that the TAB heterostructure had been successfully synthesized during the process of high temperature calcination [44].

The selected electron diffraction pattern (the insert) in Figure 2e also indicated that the synthesized TAB were polycrystalline. Subsequently, the TAB@S/PPy sample was obtained after sulfur loading and PPy coating. The SEM and TEM images of the TAB@S sample are shown in Figure 2b, and show that after sulfur loading, the original morphology of the TAB sample was maintained, but the pores in TAB sample were evenly filled with sulfur; after PPy coating, it can be seen that a thin layer was evenly distributed on the surface of the TAB@S composite, which did not change the morphology of the composite (Figure 2c,f). PPy formed a conductive layer on the material surface (Figure S2), which was conducive to improving the conductivity of the TAB@S composite. Moreover, the high-angle annular dark field scanning TEM (HAADF-STEM) images and corresponding EDS analysis showed that C, O, Ti and S elements were evenly distributed in the TAB@S/PPy sample, as shown in Figure 2g.

**Figure 3:**
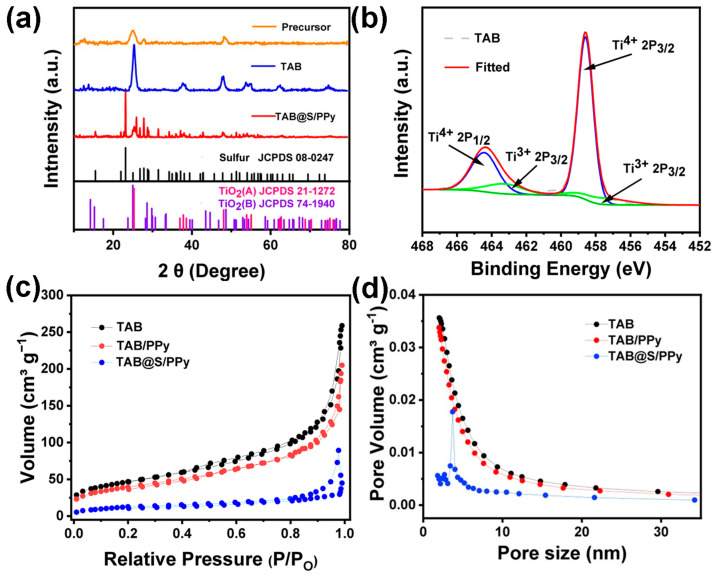
(**a**) XRD patterns of TiO_2_ precursors (JCPDS 74-1940), TAB (JCPDS No. 08-0247) and TAB@S/PPy (JCPDS No. 08-0247); (**b**) XPS spectra of TAB, N_2_ adsorption–desorption isotherms and corresponding (**c**) BET specific surface area and (**d**) pore size distribution of TAB, TAB@S and TAB@S/PPy samples.

In order to ensure the successful preparation of TAB, X-ray diffraction (XRD) tests were carried out for TiO_2_ precursors before and after annealing. The XRD spectra of the precursor in Figure 3a shows that the TiO_2_ precursor was amorphous, mainly due to the presence of P_123_, consistent with the SEM and TEM images. However, the XRD of TiO_2_ after annealing showed strong crystallization peaks, and the diffraction peaks centered at 14.21°, 28.61° and 43.31° corresponded to the TiO_2_ (B) standard card JCPDS 74-1940. The peaks at 25.28°, 36.94°, 48.04°, 62.68° and 74.02° corresponded to the (101), (103), (200), (204) and (107) crystal planes of TiO_2_ (A) (JCPDS 21-1272), which was consistent with the HRTEM in Figure 2e. The increased peak strength and absence of other miscellaneous peaks indicated that the prepared TAB had high crystallinity and was free of impurities. After sulfur loading, the characteristic peak of S was observed, proving that the sulfur was successfully melted into the pores of TAB, which was in good agreement with the SEM images. After the coating of PPy, there was no significant change in the XRD pattern of TAB@S/PPy, which was because the coated PPy layer was very thin. In Figure 3b, the XPS analysis of the TAB showed that Ti mainly existed in the form of Ti^4+^ in TAB, but there was also a small amount of Ti^3+^ on the surface, which was possibly caused by the P_123_-derived carbon as the reducing agent during the annealing process. The existence of Ti^3+^ would also introduce a certain amount of oxygen vacancy, which is beneficial to the adsorption of LiPSs [52].

The pore size distribution and specific surface area of TAB, TAB/PPy and TAB@S/PPy (Figure 3c,d) were obtained using nitrogen adsorption/desorption (BET) analysis. The BET curves of TAB in Figure 3c show type III characteristics, with an average BET specific surface area of 167.72 m^2^ g^−1^. The large specific surface area can provide abundant space for the adsorption of polysulfides, inhibiting their shuttle effect. Figure 3d depicts the pore size in TAB as predominately less than 15 nm, indicating that the pores in TAB are mainly mesoporous, in that a lot of mesoporous pores can not only provide a large amount of volume to store sulfur and relieve the volume expansion during cycling, but also physically retard the shuttling of the polysulfide [51]. According to Figure 3c,d, the coating of PPy had little effect on the pore size distribution and specific surface area of TAB. After sulfur-loading, the average specific surface area of TAB@S/PPy dropped to 39.00 m^2^ g^−1^, and the average pore size decreased to only 5 nm, indicating that sulfur molecules had been uniformly diffused to TAB mesoporous, but the TAB host still had a part of space that could be used to relieve the sulfur volume expansion in the process of charging and discharging processes, which was conductive to improving the stability of the cathode. Moreover, the sulfur content in positive electrodes is an important parameter, so thermogravimetric analysis tests were carried out to examine the content of S in TAB@S/PPy. As shown in Figure S3, TAB@S/PPy had a mass loss of about 61.53%, while TAB/PPy had a mass loss of about 7.76% for samples heated to 300 °C. Therefore, it can be calculated that the sulfur content in the TAB@S/PPy composite material was about 53.77%.

**Figure 4:**
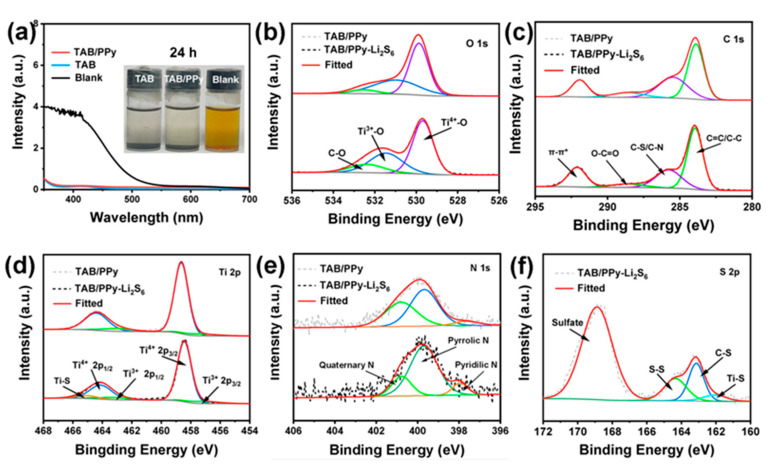
(**a**) UV-vis spectra of TAB and TAB/PPy after adsorbing Li_2_S_6_, and the optical photographs of the blank sample, TAB and TAB/PPy (insert). High resolution XPS before and after adsorption of Li_2_S_6_ by TAB/PPy: (**b**) O 1s, (**c**) C 1s, (**d**) Ti 2p, (**e**) N1s and (**f**) S 2p.

To investigate the adsorption ability of Li_2_S_6_ for TAB and TAB/PPy samples, optical visualization experiments and UV-vis spectroscopy tests were performed (Figure 4a). According to previous reports, the 0.5 M Li_2_S_6_ solution was selected to replace the LiPSs. The same mass of TAB and TAB/PPy were added to the solution. By observing the decolorization degree of different Li_2_S_6_ solutions, the adsorption capacity of the sulfur host material TAB/PPy for LiPSs was determined. After 24 h, compared with the blank Li_2_S_6_ electrolyte, the color of the Li_2_S_6_ electrolyte with TAB and TAB/PPy samples changed from yellow to transparent (insert), indicating that TAB and TAB/PPy have strong adsorption capacities for LiPSs. To further confirm the adsorption ability of TAB/PPy, we performed UV-vis tests on the three electrolytes after 24 h of rest (Figure 4a).

In the UV-vis spectrum, the characteristic peaks of Li_2_S_6_ in the 400–500 nm region completely disappeared, while the blank sample showed a strong characteristic peak in the region of 350~450 nm, which was in good agreement with the previously reported paper [41,42,43]. This strong background peak was mainly due to the high concentration of Li_2_S_6_ [12]. These results indicate that there was strong chemical adsorption and interaction between TAB@S/PPy and LiPSs, which is conducive to TAB’s adsorption of LiPSs [40].

For the purpose of determining the surface composition and chemical state in TAB/PPy and further illustrating the chemical relationship between TAB/PPy and LiPSs (denoted as TAB/PPy-Li_2_S_6_), XPS tests of TAB/PPy adsorbed powder before and after the adsorption of Li_2_S_6_ were performed. The XPS spectra (Figure 4 and Figure S4) show the chemical states of O, C, Ti, N and S in TAB/PPy, in which there were three significant peaks (Figure 4b), centered at the binding energies of 529.9, 531.5 and 532.6 eV, corresponding to Ti^4+^-O, Ti^3+^-O and C-O, respectively, with Ti^3+^ mainly coming from the annealing process [53,54]. The fitting of the C 1s region (Figure 4c) indicated the presence of multiple chemical forms of carbon. Specifically, the binding energy of 289.2 eV corresponded to the O-C=O group, while peaks of C-S or C-N bonds could be observed at 285.5 eV, indicating an interaction between S and PPy [55,56]. The peaks of the Ti 2p XPS spectrum in Figure 4d depict the peaks centered at 464.4, 463.1, 458.7 and 457.1 eV, corresponding to Ti^4+^ 2p_1/2_, Ti^3+^ 2p_1/2_, Ti^4+^ 2p_3/2_ and Ti^3+^ 2p_3/2_, respectively [57]. The presence of Ti^3+^ further proves many O vacancies existing in TAB. Importantly, a Ti-S bond corresponding to 465.1 eV in the Ti 2p XPS spectra of TAB/PPy-Li_2_S_6_ reveals the chemisorption between TiO_2_ and S, which could effectively restrain the “shuttle effect” of LiPSs through a Lewis acid–base interaction [57]. After the adsorption experiment, the peaks in Ti 2p of the TAB/PPy-Li_2_S_6_ sample depicted a slight shift to lower binding energies compared with the pristine TAB/PPy sample, which was mainly due to the strong chemistry between Ti atom/O vacancies in TAB and the S atom in Li_2_S_6_ [58]. In Figure 4e, the N 1s spectrum was fitted to obtain three peaks corresponding to quaternion nitrogen (400.8 eV), pyrrolic nitrogen (399.7 eV) and pyridine nitrogen (397.9 eV) [50]. After Li_2_S_6_ adsorption, the N peaks of the TAB/PPy sample underwent a slight shift to lower binding energies, indicating that N played a definite role in the adsorption of LiPSs [40]. As shown in Figure 4f, the three distinct peaks at 164.5, 163.2 and 161.9 eV could be assigned to S-S, C-S and Ti-S bonds (Figure 4f), respectively, which further indicated that TAB and PPy in TAB/PPy have strong chemical interactions with LiPSs [59,60]. The above XPS results confirmed the strong adsorption and chemical interaction between TAB/PPy and LiPSs species, which was consistent with the optical adsorption experiment and UV-vis adsorption spectra.

**Figure 5:**
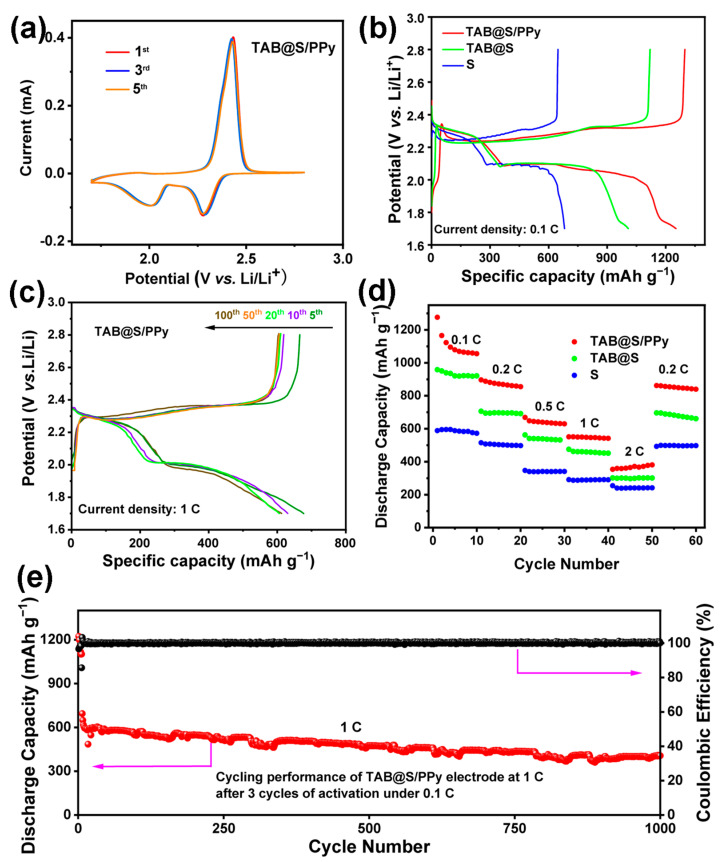
Electrochemical properties of the cells with TAB@S/PPy, TAB@S and sulfur cathodes. (**a**) Cyclic voltammetry curves of the Li-S battery with TAB/S@PPy. (**b**) Initial charge-discharge curves of the batteries with TAB@S/PPy, TAB@S and sulfur electrodes at 0.1 C. (**c**) Voltage profiles of the TAB@S/PPy cathode at 1 C. (**d**) Rate performance of TAB@S/PPy, TAB@S and sulfur cathodes at 0.1, 0.2, 0.5, 1 C and 2C, (**e**) long-cycling performance of Li-S batteries with the TAB@S/PPy cathode at 1 C.

The electrochemical performances of the Li-S batteries with TAB@S/PPy, TAB@S and sulfur electrodes were further investigated (Figure 5). The cyclic voltammetric (CV) curves of the first five cycles of the cell with TAB@S/PPy electrode are shown in Figure 5a. In the first cathodic scan, two distinct peaks were observed at around 2.32 V and 2.02 V, which could be attributed to the conversion of sulfur to long-chain LiPSs (Li_2_S_n_, 4 ≤ n ≤ 8) and further transformation into short-chain Li_2_S_2_ or Li_2_S, respectively [39]. During the following cycles in CV curves, no obvious change in the intensity and position of characteristic peaks could be observed, indicating the excellent reversibility of the battery with the TAB@S/PPy cathode. Compared with the battery with the sulfur cathode (Figure S5), the CV curve of the battery with the TAB@S/PPy displayed a larger current density, indicating that the battery with the TAB@S/PPy cathode could provide a greater capacity. Figure 5b shows the initial galvanostatic charge–discharge curves of Li-S batteries with TAB@S/PPy, TAB@S and sulfur electrodes at 0.1 C. The cell with the TAB@S/PPy cathode could deliver an initial discharge capacity of TAB@S/PPy of 1250.4 mAh g^−1^, much higher than that with TAB@S and sulfur cathodes. These batteries exhibited two distinct discharge plateaus and one charging plateau, which corresponded to two reduction peaks and one oxidation peak in the CV curves, respectively [61]. As shown in Figure 5b, the Li-S battery with TAB@S/PPy had a higher capacity than that with TAB@S and sulfur electrodes, which could be ascribed to the PPy on the surface of TAB being able to increase the electronic conductivity of the electrode. The charge–discharge curves of the TAB@S/PPy cell for the first 100 cycles at 1 C were depicted in Figure 5c. After five cycles, the cell could display an outstanding discharge capacity of 662.8 mAh g^−1^. After that, the charging and discharging plateaus of TAB@S/PPy cell did not change obviously, and the battery could deliver a discharge capacity of ~600 mAh g^−1^ at 1 C after 100 cycles, which indicated that the Li-S battery with the TAB@S/PPy electrode had excellent cycling stability and reversibility.

The rate capabilities of the Li-S battery with TAB@S/PPy, TAB@S and sulfur electrodes at various rates are depicted in Figure 5d. The specific discharge capacity of the TAB@S/PPy battery (1250.4 mAh g^−1^) was higher than that of the TAB@S and sulfur cathodes, at 0.1 C, and when the current density increased to 2 C the specific discharge capacity of nearly 380.4 mAh g^−1^ could still be maintained. In contrast, the battery with the sulfur cathode could only deliver a capacity of 254 mAh g^−1^. When re-switched to 0.2 C, the discharge capacity of TAB@S/PPy could still be restored to the same degree as before, showing good reversibility. In order to test the long cycle performance of the full battery composed of the TAB@S/PPy composite, we carried out the activation process at a low current of 0.1 C, and then tested the cyclic performance at 1 C (Figure 5e). After 1000 cycles, it still could deliver a high specific discharge capacity of 406.1 mAh g^−1^, corresponding to the capacity decay rate of only about 0.042% per cycle. The excellent cycling stability may be attributed to the incorporation of TAB and PPy, which provided abundant active sites for the adsorption of LiPSs, as well as improved electrode electronic conductivity, successfully inhibiting the shuttle effect of LiPSs [62,63].

We determined the performance of different previous TiO_2_-based lithium-sulfur batteries in Table S1 [54,64,65,66,67]. In order to make the comparison more meaningful, the sulfur content, initial capacity, rate performance and cyclic stability of the different cathodes were compared, respectively. Through comparison, it was found that the initial capacity and cyclic stability of the TAB@S/PPy electrode were excellent, especially the cyclic stability, which was superior to other works. This was mainly due to the unique structure of the TAB/PPy composite and the excellent electrical conductivity of PPy.

**Figure 6:**
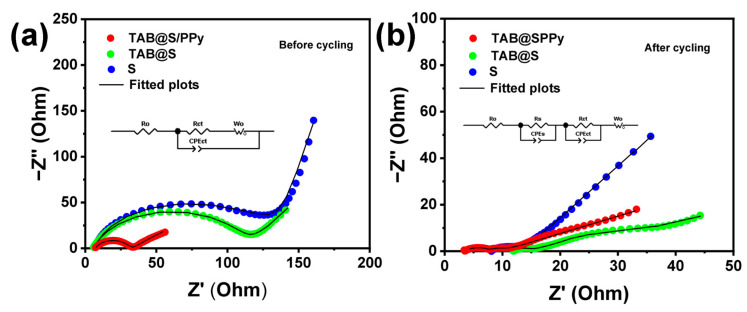
EIS curves for cells with TAB@S/PPy, TAB@S and sulfur cathodes (**a**) before and (**b**) after 200 cycles at 0.5 C, and insets are equivalent circuits.

The electrochemical impedance spectroscopy (EIS) plots before and after 200 cycles at 0.5 C of the battery with TAB@S/PPy, TAB@S and sulfur cathodes are shown in Figure 6 at an open-circuit voltage measured from 100 kHz to 10 mHz with an amplitude of 5 mV. The EIS plots of the battery present a typical semicircle at high and middle frequencies and a linear curve at low frequencies. Before cycling, the charge transfer resistance (Rct) of the cell with the TAB@S/PPy electrode was tested to be 33.85 Ω, which was much lower than that of the cells with TAB@S (100.08 Ω) and sulfur cathodes (109.3 Ω). After cycling, the Rct of the TAB@S/PPy battery decreased to 3.67 Ω, facilitating the rapid transfer of electrons and ions, which was still much smaller than that of the battery with TAB@S (10.1 Ω) and sulfur cathodes (150.78 Ω), as shown in Table S2 [54]. The intercept of the curve on the horizontal axis represents the internal resistance of the battery, and the smaller intercept of the TAB@S/PPy electrode corresponds to the smaller internal resistance of the battery, which also explains the better performance of the TAB@S/PPy electrode during cycling. In addition, the introduction of conductive PPy further reduced the electrode resistance and accelerated the transmission of lithium ions, further enhancing electrochemical reaction dynamics.

## 3. Materials and Methods

### 3.1. Preparation of Anatase/Bronze TiO_2_ (TAB)

The TAB heterostructure was prepared using the hydrothermal method and subsequent annealing process, as described elsewhere [38]. First, 0.2 g P123 and 3 g of anhydrous ethanol were thoroughly mixed for 10 min to form solution A. Second, 0.2 g tetrabutyl titanate and 0.74 g concentrated HCl were mixed and stirred for 10 min to produce solution B, and then the solution was added to solution B, forming solution C, the color of which turned yellow. Third, 35 mL glycol was added to solution C and stirred for 0.5 h. That solution was transferred to autoclave and heated under 150 °C for 20 h. The hydrothermal products were centrifuged and cleaned with distilled water and anhydrous ethanol repeatedly 3 times, and dried under 150 °C for 20 h. The obtained dried block solid was ground to obtain the precursor. After that, the precursor was placed in a tubular furnace and heated at 600 °C with the heating rate of 5 °C·min^−1^ in the environment of argon gas. The TAB sample was obtained after being kept for 4 h and naturally cooled to room temperature.

### 3.2. Preparation of TAB@S

Totals of 15 mL CS_2_ and 6 mL DMF were mixed to form a solution. Then, 0.12 g TAB and 0.28 g nano sulfur were added into the above mixed solution, and ultrasound was applied for 0.5 h. The mixture was then stirred at 45 °C for 12 h and washed with distilled water and anhydrous ethanol two times, respectively, and dried in a vacuum drying oven at 60 °C for 4 h. Finally, the pulverized solids were heated in a tubular furnace at the heat-up rate of 5 °C·per min to 155 °C for 12 h and 220 °C for 30 min, respectively, in an Ar atmosphere. After cooling, TAB@S sample was obtained.

### 3.3. Preparation of TAB@S/PPy

A 3 mL quantity of anhydrous ethanol was added into 27 mL distilled water, and then 0.3 g TAB@S, 0.02 g sodium dodecyl sulfate and 10 μL triton were mixed into the above solution and stirred for 0.5 h. After that, 5 μL pyrrole and 10 mL 0.1 M (NH_4_) S_2_O_3_ were added to that solution and stirred in ice bath for 4 h. Then, the product was washed twice with deionized H_2_O and C_2_H_5_OH. TAB@S/PPy composites were obtained after vacuum drying at 60 °C for 12 h.

### 3.4. Visible Polysulfide Adsorption Experiment

The solution of 0.5 M Li_2_S_6_ was synthesized by using a nominal stoichiometry method. Typically, a mixture of S powder and lithium sulfide (Li_2_S) was dissolved in 1, 3-dioxane (DOL): 1, 2-dimethoxy-ethane (DME) (*v*:*v* = 1:1) and magnetically stirred at 60 °C for 24 h. Then, 2 mmol L^−1^ Li_2_S_6_ solution was prepared by diluting 0.5 M Li_2_S_6_ solution with equal volume of DOL/DME. Then, 15 mg TAB/PPy was added to 3 mL 2 mmol L^−1^ Li_2_S_6_ solution. The color change of the Li_2_S_6_ solution can be observed within a few minutes. The supernatant was detected with the ultraviolet spectrum, and the precipitated products were dried for XPS analysis. The above sample preparation was carried out in an Ar-filled glove box (H_2_O and O_2_ were both less than 0.1 ppm).

### 3.5. Material Characterization

The phase and composition of the prepared materials were analyzed with XRD (Bruker D8 ADVANCE, Cu Kα source, Bruker, Billerica, MA, USA). The crystal structure and morphology of the materials were characterized with scanning electron microscopy (SEM) (JSM-5610LV, JEOL, Akishima, Japan) and transmission electron microscopy (TEM) (JSM-2100F, 200 kV, Hitachinaka, Naka, Japan). The content of sulfur in the composites was calculated with thermogravimetric analysis (NETZSCH STA 409, Netzsch, Selb, Germany) under N_2_ atmosphere. The state of the elements and the surface composition of the compounds were analyzed with X-ray photoelectron spectroscopy (PHI 5000 VersaProbe II, Physical Electronics, Inc., Chanhassen, MN, USA), and the binding energies were referenced to the C1s line at 284.8 eV from adventitious carbon The adsorption effect of the composites on sulfide was tested with the UV-visible spectrum (Shimadzu, Japan). The specific surface area and pore size distributions of the composites were measured using a fully automated specific surface and porosity analyzer (Mac ASAP2460, Norcross, GA, USA).

### 3.6. Cell Assembly and Electrochemical Performance Tests

For the cathode slurry preparation, TAB@S/PPy (70 wt%), Super P (20 wt%) and polyvinylidene fluoride (PVDF) (10 wt%) were thoroughly mixed. Then, N-methyl-2-pyrrolidone (NMP) was added to the above mixture. The slurry was evenly applied on the carbon-coated aluminum foil with a scraper and dried for 12 h under 60 °C under vacuum. Then, the dried electrode plate was cut into a diameter of 12 nm. The loading of TAB@S/PPy in the positive electrode of the coin cell was 1.0–1.5 mg cm^−2^. The lithium foil as the negative electrode and the Celgard 2500 as the separator were used for the 2032 coin cells assembly. The electrolyte was 1 M lithium difluoromethane sulfonimide (LiTFSI) + dioxolame (DOL): diethyl carbonate (DME) (1:1 in volume) added to 1% LiNO_3._ LiNO_3_ alters the solvation structure of Li+, which has a profound impact on the electrochemical performance of batteries [68]. Then, 40 μL of electrolyte was added to each cell (Figure S6) [69]. LAND CT2001A battery test system was used for the constant current charge–discharge test, and the voltage window was 1.7–2.8V. The Li-S batteries were pre-cycled three times at a low current density (0.1 C) to activate the electrodes prior to a constant-current charge–discharge test. The electrode preparation and battery assembly process of the original S electrode was consistent with that of TAB@S/PPy sample. Cyclic voltammetry (CV) tests with a scanning rate of 0.1 mV s^−1^ and electrochemical impedance spectroscopy (EIS) measurements with a frequency ranging from 100 kHz to 0.01 Hz were performed at the electrochemical workstation (Chenhua CHI 660E, Shanghai, China). In this work, the specific capacity value was calculated based on the mass of S.

## 4. Conclusions

In summary, the porous TAB@S/PPy composite material was synthesized with hydrothermal and subsequent annealing treatment, followed by sulfur loading, and coated with a thin PPy layer. Due to its unique porous heterostructure, TAB/PPy not only has enough volume to hold sulfur and relieve volume expansion during cycling, but can also physically and chemically adsorb polysulfides and inhibit their shuttle effect. In addition, the heterostructure of TAB and the conductive PPy coating layer can promote Li^+^ transport, greatly improving the conductivity of the electrode. Consequently, the battery using TAB@S/PPy as the cathode obtained a high initial capacity (1250.4 mAh g^−1^ at 0.1 C), long cycle life and excellent cycle stability (the average capacity decay rate of per cycle was only about 0.042% after 1000 cycles at 1C). The work provides a new strategy for the design of high-performance Li-S battery cathode materials.

## Data Availability

The data are contained within this article.

## Supplementary Materials

The following supporting information can be downloaded at: https://www.mdpi.com/article/10.3390/molecules28114286/s1, Figure S1 SEM image of TiO_2_ precursor; Figure S2 TEM image of TiO2@S/PPy; Figure S3 TGA of pure PPy, S@PPy, TAB@PPy and TAB@S/PPy; Figure S4 XPS survey spectrum of TAB/PPy before and after adsorption of Li2S6; Figure S5 CV profiles of pristine S cathode and TAB@S/PPy cathode at a scan rate of 0.1 mV S^−1^; Figure S6 Comparison of cycling performance of Li-S batteries with different E/S at 1C; Table S1 Electrochemical performance of the Li-S batteries with TiO_2_-based cathode materials in previous reported literatures [54,60,64,65,66,67]; Table S2 Parameters of the equivalent circuit (Figure S4) to reproduce the Nyquist plots of pure S, TAB@S, and TAB@S/PPy in Figure 6.

## Sample Availability

Samples of anatase/bronze TiO_2_ and anatase/bronze TiO_2_/polypyrrole composites are available from the authors.
