# Supplementary material for: Efficient Regulation of Polysulfides by Anatase/Bronze TiO2 Heterostructure/Polypyrrole Composites for High-Performance Lithium-Sulfur Batteries
Source: Molecules. 2023 May 23;28(11):4286. doi: 10.3390/molecules28114286 (PMC10254138; doi:10.3390/molecules28114286)
Supplement: Supplementary file 1 [file molecules-28-04286-s001.zip › molecules-2346603-supplementary.pdf]

Supporting Information

Efficient Regulation of Polysulfides by Anatase/bronze TiO_2 heterostructure/Polypyrrole for High-Performance Li-S Batteries

Jing Liu [1], Yong Liu [1,*], Tengfei Li [1], Longlong Liang [1], Sifan Wen [1], Yue Zhang [1], Guilong Liu [2], Fengzhang Ren [1] and Guangxin Wang [1,*]

[1] School of Materials Science and Engineering, Henan University of Science and Technology, Luoyang 471023, China; liuliu158600@163.com (J.L.); fei1281156656@163.com (T.L.)

[2] Key Laboratory of Function-Oriented Porous Materials of Henan Province, College of Chemistry and Chemical Engineering, Luoyang Normal University, Luoyang 471934, China

* Correspondence: liuyong209@haust.edu.cn (Y.L.); wanggx2016@126.com (G.W.)

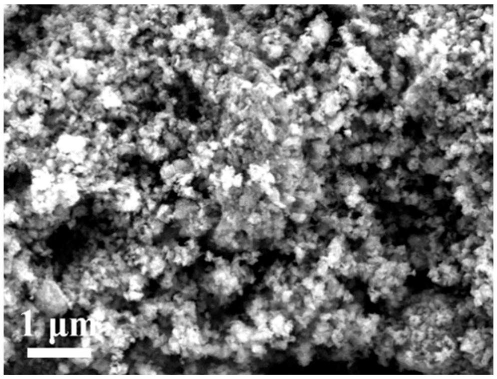

Figure S1 SEM image of TiO_2 precursor.

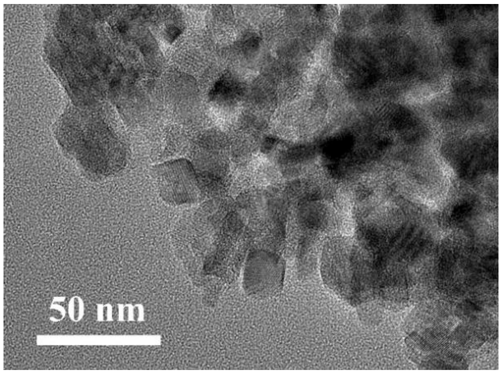

Figure S2 TEM image of TiO_2@S/PPy.

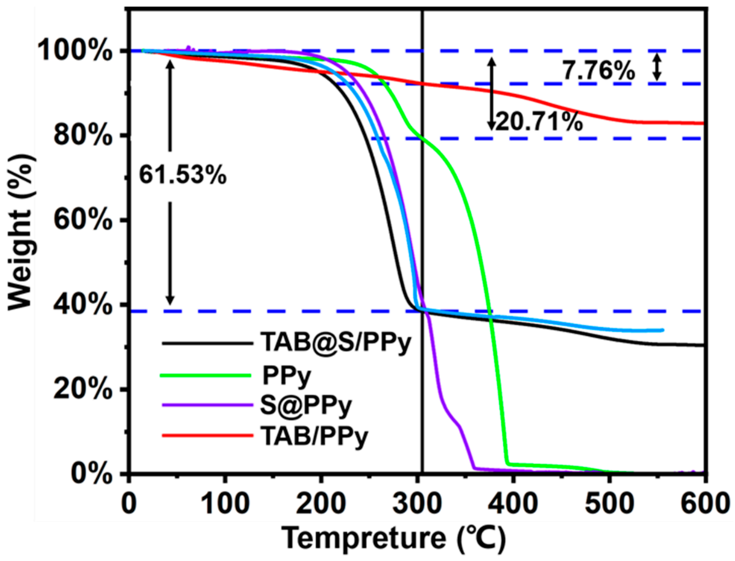

Figure S3 TGA of pure PPy, S@PPy, TAB@PPy and TAB@S/PPy.

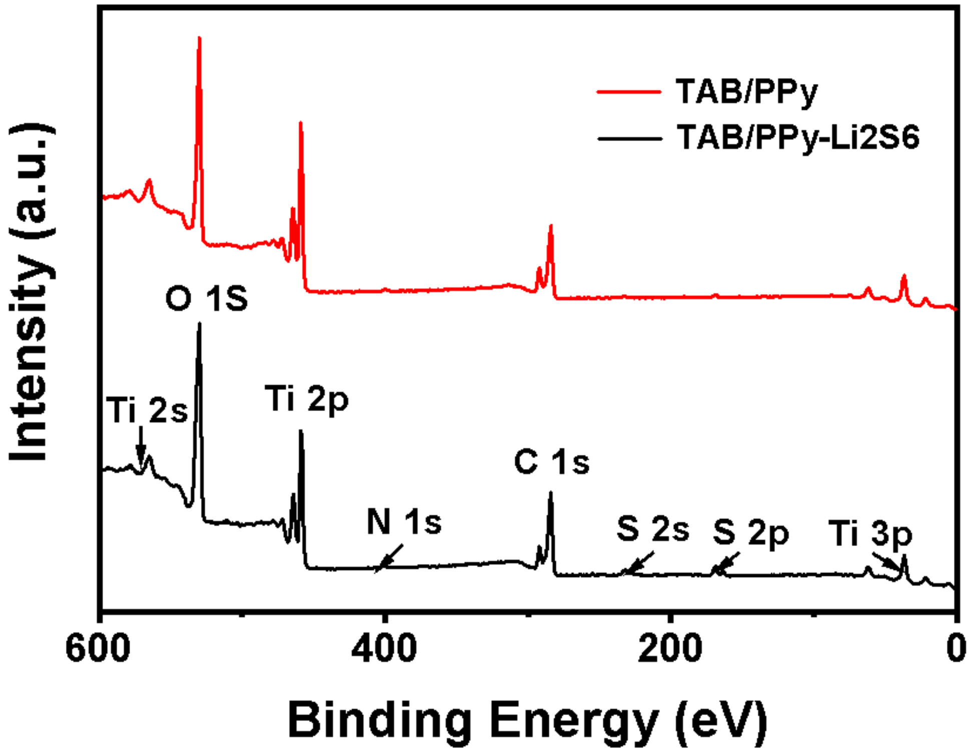

Figure S4 XPS survey spectrum of TAB/PPy before and after adsorption of Li_2S_6.

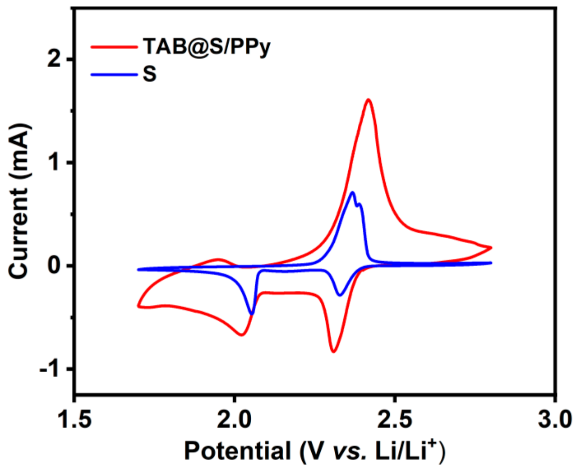

Figure S5 CV profiles of pristine S cathode and TAB@S/PPy cathode at a scan rate of 0.1 mV S^{-1}.

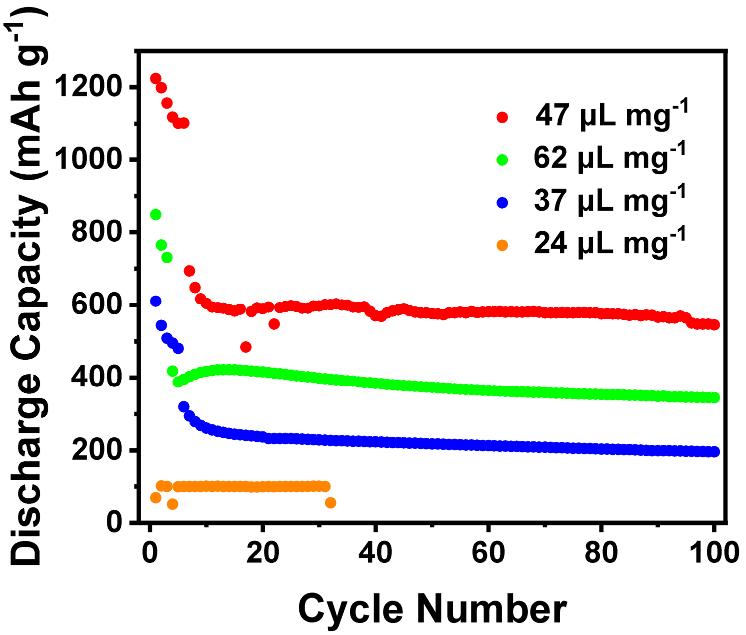

Figure S6 Comparison of cycling performance of Li-S batteries with different E/S at 1C.

According to the thermogravimetric analysis results, the sulfur content of TAB@S/PPy is 53.77%, and the loading of TAB@S/PPy is 1-1.5 mg cm^{-2}. When the loading mass of TAB@S/PPy is 1.5 mg cm^{-2}, and the 40 μL electrolyte was used for the Li-S battery assembly, corresponding to an E/S of 43.8 μL mg^{-1}. We also assemble the battery with 30 μL electrolyte, which has an E/S of 32.9 μL $mg^{-1.}$ Similarly, we also assemble the battery with a 20 μL electrolyte with an E/S of 21.9 μL mg^{-1}. The cycling performance of batteries with different E/S is shown in the figure below. As shown in the figure below, with the decrease of E/S, the battery capacity is gradually decrease. So, we chose 40 μL electrolyte with an E/S of 43.8 μL mg^{-1} to assemble the batteries.

Table S1 Electrochemical performance of the Li-S batteries with TiO_2-based cathode materials in previous reported literatures..

Materials	Sulfur content/Loading	Initial discharge specific capacity	Rate performance	Cycling stablity	Ref.
b-TiO_2/S@PPy	59.9 wt%/ 1.5-1.8 mg cm^{-2}	1374 mAh g^{-1} at 0.1C	725.0 mAh g^{-1} at 2C	910 mAh g^{-1} after 200 cycles at 0.1C, 0.169% per cycle	[54]
S@TiO_2/PPy	72.4 %/-	1385.9 mAh g^{-1} at 0.2 C	505.2 mAh g^{-1} at 2 C.	459.6 mAh g^{-1} after 500cycles at 1 C, 0.047 % per cycle	[60]
TiO_2/S@PPy	72.61%/-	1013.7 mAh g^{-1} at 0.1 C	402.0 mAh g^{-1} at 2C	567.0 mAh g^{-1} after 300 cycles at 0.5 C, 0.103% per cycle	[64]
H-TiO_2/S	~67 wt.%/-	902 mAh g^{-1} at 0.5 C	~579 mAh g^{-1} at 2 C	688 mAh g^{-1} after 200cycles at 0.5 C, ~0.399% per cycle	[65]
S/PPy/TiO_2 NTs-300	~66.2 wt.%/-	1350 mAh g^{-1} at 0.05 C	500 mAh g^{-1}at 1 C	1150 mAh g^{-1} after 100 cycles at 0.1 C, 0.37% per cycle	[66]
H-TiOx@S/PPy	~66.33 wt.%/ 1.0 mg cm^{-2}	1050 mAh g^{-1} at 0.5 C	726 mAh g^{-1} at 1 C	411.7 mAh g^{-1} after 1000 cycles at 1 C, 0.0406% per cycle	[67]
TAB@S/PPy	53.77%/1-1.5 mg cm^{-2}	1250.4 mAh g^{-1} at 0.1 C	662.8 mAh g^{-1} at 1C	406.1 mAh g^{-1} after 1000 cycles at 1 C, 0.042% per cycle	This work

Table S2 Parameters of the equivalent circuit (Fig. S4) to reproduce the Nyquist plots of pure S, TAB@S, and TAB@S/PPy in Fig. 6, before and after 200 cycles. Ro is the ohmic resistance of the cell including electrolyte and electrode. Rs is the resistance associated to the solid-electrolyte interface (SEI). Rct , CPE ct are associated to the charge-transfer.

Condition	Electrodes	Ro [Ω]	R_S [Ω]	R_{ct} [Ω]
Before cycling	Pristine S	6.56	-	109.30
	TAB@S	5.54	-	100.08
	TAB@S/PPy	7.07	-	33.85
After cycling	Pristine S	7.37	6.41	150.78
	TAB@S	11.99	2.01	10.10
	TAB@S/PPy	3.56	1.89	3.67

References:

54 Wu, T.; Sun, G.R.; Lu, W.; Zhao, L.P.; Mauger, A.; Julien, C.M.; Sun, L.Q.; Xie, H.M.; Liu, J.H. A polypyrrole/black-TiO2/S double-shelled composite fixing polysulfides for lithium-sulfur batteries. *Electrochim. Acta* **2020**, *353*, 11.

60. Wu, J.; Li, S.Y.; Yang, P.; Zhang, H.P.; Du, C.; Xu, J.M.; Song, K.X. S@TiO_2 nanospheres loaded on PPy matrix for enhanced lithium-sulfur batteries. *J. Alloys Compd.* **2019**, *783*, 279–285.

64. Song, H.S.; Yuan, H.L.; Chen, H.Z.; Tang, A.P.; Xu, G.R.; Liu, L.H.; Zhang, Z.; Kuang, Q.J. Synthesis of TiO_2/S@PPy composite for chemisorption of polysulfides in high performance Li-S batteries. *J. Solid State Electrochem.* **2020**, *24*, 997–1006.

65. Yuan, C.; Zhu, S.; Cao, H.; Hou, L.; Lin, J. Hierarchical sulfur-impregnated hydrogenated TiO_2 mesoporous spheres comprising anatase nanosheets with highly exposed (001) facets for advanced Li-S batteries. *Nanotechnology* **2016,** *27*, 045403.

66. Zhao, Y.; Zhu, W.; Chen, G.Z.; Cairns, E.J. Polypyrrole/TiO_2 nanotube arrays with coaxial heterogeneous structure as sulfur hosts for lithium sulfur batteries. *J. Power Sources* **2016**, *327*, 447–456.

67. Chen, G.L.; Zhong, W.T.; Li, Y.S.; Deng, Q.; Ou, X.; Pan, Q.C.; Wang, X.W.; Xiong, X.H.; Yang, C.H.; Liu, M.L. Rational Design of TiO-TiO2 Heterostructure/Polypyrrole as a Multifunctional Sulfur Host for Advanced Lithium-Sulfur Batteries. *Acs Appl. Mater. Interfaces* **2019**, *11*, 5055–5063.
